# Optimizing precision in ICU arterial access: the impact of ultrasound-guided dynamic needle tip positioning

**DOI:** 10.3389/fmed.2025.1625108

**Published:** 2025-07-31

**Authors:** Xiuqin Chen, Yongrui Wu, Li Zhang, Ying Zhou, Hui Fang

**Affiliations:** ^1^Intensive Care Unit, Taihe County People’s Hospital, Fuyang, Anhui, China; ^2^Medical School, Fuyang Normal University, Fuyang, Anhui, China

**Keywords:** ultrasonography, catheterization, critical care, shock, intraoperative complications

## Abstract

**Objective:**

This study aims to evaluate the efficacy of ultrasound-guided dynamic needle tip positioning (DNTP) in arterial puncture and catheterization among ICU patients.

**Methods:**

A cohort of 55 patients in shock, requiring arterial catheterization in the ICU from April 2020 to July 2024, was enrolled and randomly stratified into groups based on distinct ultrasound-guided puncture techniques. Of these, 27 patients who underwent the ultrasound-guided direct entry (UGDE) method were designated as the control group, while 28 patients who received arterial catheterization via the ultrasound-guided DNTP technique were assigned to the observation group. Comparative analyses were conducted on the first-attempt puncture success rate, first-attempt catheterization success rate, puncture duration, and incidence of puncture-related complications between the two groups.

**Results:**

The first-attempt puncture success rate did not significantly differ between the observation and control groups (*P* = 0.98). However, the observation group exhibited a higher first-attempt catheterization success rate (*P* = 0.049), an extended puncture duration (*P* < 0.001), and a reduced overall incidence of puncture-related complications (*P* = 0.049) in comparison to the control group.

**Conclusion:**

The application of ultrasound-guided DNTP methodology demonstrates statistically significant improvement in arterial catheterization procedural success rates concurrent with a marked reduction in iatrogenic vascular access complications.

## Introduction

Arterial puncture and catheterization are standard procedures in the fields of anesthesiology, emergency medicine, and critical care medicine. These techniques facilitate continuous monitoring of arterial blood pressure and allow for repeated arterial blood sampling, proving particularly beneficial for critically ill patients in intensive care units (ICUs) or those undergoing major surgeries under general anesthesia. By offering a dynamic and precise assessment of blood pressure levels, arterial catheterization serves as a crucial hemodynamic parameter that informs clinical decision-making (1–3). Traditionally, clinicians have performed arterial punctures using the landmark-guided technique, which relies on the palpation of arterial pulses and empirical experience. However, this method is susceptible to failure due to various factors, often requiring multiple attempts. These repeated punctures not only extend the duration of the procedure and increase patient discomfort but may also adversely affect subsequent clinical management (4–6). With the ongoing advancements in ultrasound technology and its widespread application in clinical diagnostics and treatment practices, nurses in the ICU department at Taihe County People’s Hospital have adopted ultrasound-assisted arterial puncture techniques. Under ultrasound guidance, operators can visualize arterial vascular structures in real-time, facilitating puncture procedures with direct visualization and addressing the limitations associated with the traditional palpation method. Currently, ultrasound-guided arterial puncture techniques encompass two primary approaches: the long-axis in-plane and the short-axis out-of-plane methods. Despite these advancements, there is considerable potential for enhancing procedural success rates, as current first-attempt success rates for these techniques range from 67.6 to 85.9% (7–9). Within the short-axis out-of-plane approaches, techniques such as ultrasound-guided dynamic enhancement (UGDE) and dynamic needle tip positioning (DNTP) have gained widespread adoption. Notably, DNTP is a modified technique based on the short-axis out-of-plane approach, where needle advancement is synchronized with probe manipulation to maintain the needle tip’s centering within the vascular lumen. This method significantly improves arterial catheterization success rates while minimizing complications (10–12). In light of this, the present study aims to evaluate the efficacy of ultrasound-guided DNTP for arterial catheterization in a cohort of 55 critically ill patients experiencing shock.

## Materials and methods

### General materials

A cohort of 55 patients experiencing shock and requiring arterial catheterization in the ICUs was analyzed between April 2020 and July 2024. This research was approved by the Taihe county people’s hospital and written informed consent was obtained from all subjects participating in the trial. These patients were randomly divided into two groups using an ultrasound-guided method. The control group consisted of 27 patients receiving UGDE (13 males and 14 females; age range: 34–87 years, mean age: 68.59 ± 16.71 years; hospitalization duration: 6–55 days, mean duration: 14.74 ± 10.27 days; with 21 cases of septic shock and 6 cases of hemorrhagic shock). The observation group included 28 patients receiving DNTP (17 males and 11 females; age range: 45–91 years, mean age: 67.68 ± 12.73 years; hospitalization duration: 7–39 days, mean duration: 14.07 ± 7.89 days; with 25 cases of septic shock and 3 cases of hemorrhagic shock). Baseline characteristics revealed no statistically significant differences between the two groups (*P* > 0.05).

#### Inclusion criteria

(1) Patients with indications for arterial catheterization, such as those undergoing major surgery or experiencing shock. (2) Patients requiring continuous monitoring of blood pressure changes or exhibiting hemodynamic instability. (3) Patients necessitating repeated arterial blood sampling for blood gas analysis. (4) Patients with a negative result on the modified Allen test.

#### Exclusion criteria

(1) The presence of contraindications for arterial puncture and catheterization, such as a positive Allen test or coagulation disorders. (2) Patients undergoing hemodialysis with arteriovenous fistulas. (3) The presence of infection or trauma at or near the puncture site. This study was approved by the Ethics Committee of Taihe County People’s Hospital (Approval No. 2020-71), and written informed consent forms were signed by all enrolled patients or their legal guardians. Informed consent procedures were stratified by patient consciousness status: (1) conscious shock patients provided self-signed consent. (2) Those with impaired consciousness required priority consent from legal representatives (first-degree relatives or court-appointed guardians). (3) Emergency waivers were strictly limited to critically ill patients lacking contactable representatives under preapproved institutional review board protocols (FDA 21 CFR 50.24 compliance).

### Methods

All arterial cannulation procedures were conducted by ICU specialist nurses who had received training in ultrasound techniques. Prior to performing arterial cannulation, the anatomical location of the blood vessel was identified. The radial artery was selected as the puncture site for both groups in this study due to its superficial location, ease of palpation, and convenience for nursing care. Following a modified Allen’s test to confirm a negative result, Doppler ultrasound was employed to assess the presence of thrombosis and measure blood flow velocity within the vessel, thereby confirming its patency. The patient was positioned supine with the limb on the puncture side naturally abducted. The local skin at the puncture site was disinfected twice using a 0.5% iodophor solution, and a sterile drape was applied. The operator wore sterile gloves and utilized an ultrasound probe covered with a sterile protective sleeve. The operator conducted an ultrasound scan of the blood vessel in a short-axis view and centered the probe on the radial artery to identify and mark the puncture site (Supplementary Video). The specific puncture procedure is outlined as follows:

#### UGDE procedure for the observation group

(1) The operator positions the ultrasound probe over the radial artery with the left hand while holding the puncture needle in the right hand, inserting it at an angle of 30°–40°. (2) Upon observing blood return, the operator slightly depresses the puncture needle and advances it by 1–2 mm to ensure consistent blood return, subsequently withdrawing the needle while simultaneously advancing the outer cannula. (3) The degassed pressure sensor is then connected to the puncture needle and monitor to commence invasive arterial pressure monitoring.

#### Observation indicators

The DNTP method integrates the UGDE technique with dynamic needle tip positioning technology to facilitate intravascular needle guidance, enabling real-time tracking of the needle tip during the puncture process. The operator inserts the puncture needle at an angle of 30°–40° until the hyperechoic tip is visualized entering the vessel on the short-axis plane. The needle tip is subsequently adjusted to be centered within the arterial lumen. While maintaining the needle in a fixed position, the ultrasound probe is moved parallel to the proximal end until the hyperechoic tip is no longer visible in the image. With the probe held steady, the needle is advanced further until the hyperechoic tip reappears. The angle of the puncture needle is adjusted to ensure it remains centered within the ultrasound image. This procedure is repeated until the hyperechoic tip of the needle consistently appears centered within the radial artery on the short-axis image. At this juncture, the cannula is inserted, and the pressure sensor is connected to facilitate invasive arterial pressure monitoring.

This study compared the number of successful first-attempt punctures, successful first-attempt catheterizations, puncture duration, and puncture-associated complications between the two patient groups. (1) First-Attempt Puncture Success: This refers to the successful achievement of venipuncture on the initial attempt at skin penetration, characterized by the immediate return of blood at the needle tip, regardless of the final outcome of cannulation. (2) First-Attempt Cannulation Success: This denotes the successful placement of a catheter achieved through a single penetration of the skin without necessitating needle redirection. (3) Puncture Duration: This is defined as the time interval from the initial contact of the ultrasound-guided needle with the skin to the final successful cannulation. In cases involving multiple attempts, the duration is recorded up to the last successful procedure. (4) Procedure-Related Complications: These are adverse events such as hematoma formation, vascular spasm, or other iatrogenic injuries that are documented within 24 h following the procedure.

#### Data collection and quality control measures

In this study, assessors and clinical operators were separated and received standardized training, respectively, to minimize subjective bias. All procedures in both the observation group and control group were performed by two specialty-certified nurses who had completed specialized training and passed competency assessments, ensuring protocol compliance and procedural consistency.

### Statistical analysis

Statistical analysis was conducted using SPSS version 22.0 software. Continuous variables were expressed as mean ± standard deviation (SD) and analyzed using the student’s *t*-test. Categorical data were presented as numbers (percentages) and compared using the Chi-square test. A statistically significant difference was defined as *P* < 0.05.

## Results

### Baseline characteristics of the two groups

No statistically significant differences in baseline characteristics were observed between the two groups (*P* > 0.05), as summarized in Table 1.

**TABLE 1:** Comparison of general data between the two groups.

Groups		Observation group(*n* = 28)	Control group (*n* = 27)	*χ ^2^/t*	*P*
Gender [*n*, male/female]		17/11	13/14	0.875	0.35
Age [years, (x̄ ± s)]		67.68 ± 12.73	68.59 ± 16.71	0.228	0.82
APACHEII score [points, (x̄ ± s)]		18.46 ± 6.63	17.19 ± 6.47	0.7189	0.48
Shock index (x̄ ± s)		1.28 ± 0.44	1.11 ± 0.33	1.616	0.11
Length of hospital stay [days, (x̄ ± s)]		14.07 ± 7.89	14.74 ± 10.27	0.272	0.79
Type of shock [*n*, (%)]	Septic shock	25 (89.29)	21 (77.78)	0.622	0.43
	Hemorrhagic shock	3 (10.71)	6 (22.22)		

### Comparison of puncture catheterization between the two groups

No statistically significant difference was observed in the first-puncture success rate between the observation and control groups (*P* = 0.98). However, the first-catheterization success rate was significantly higher in the observation group compared to the control group (*P* = 0.049). Additionally, the puncture time was marginally longer in the observation group than in the control group (*P* < 0.001), as detailed in Table 2.

**TABLE 2:** Comparison of puncture catheterization between the two groups.

Groups	First puncture success rate [*n* (%)]	First-time catheterization success rate [*n* (%)]	Puncture duration (x ± s)
Observation group (*n* = 28)	27 (96.43)	27 (96.43)	101.33 ± 10.55
Control group (*n* = 27)	25 (92.59)	20 (74.07)	91.26 ± 8.56
χ^2^/t	0.001➀	3.874➀	3.879➁
*P*	0.975	0.049	<0.001

➀ χ^2^ value; ➁ *t*-value.

### Comparison between the puncture-related complications of two groups

The overall incidence of puncture-related complications was significantly lower in the observation group compared to the control group (*P* = 0.049), as illustrated in Table 3.

**TABLE 3:** Comparison of the incidence of puncture-related complications between the two groups.

Groups	Hematoma	Convulsion	Overall incidence rate
Observation group (*n* = 28)	0(0.00)	1(3.57)	1(3.54)
Control group (*n* = 27)	3(11.11)	4(14.81)	7(25.93)
*χ^2^*	–	–	3.874
*P*	–	–	0.049

## Discussion

Ultrasound, as a non-radiative and real-time dynamic imaging modality, facilitates healthcare professionals in the precise localization and manipulation of target structures under its accurate guidance, thereby expanding its clinical applicability. Ongoing technological advancements and innovations have enhanced ultrasound equipment and probes, resulting in clearer tissue imaging. Ultrasound-guided puncture techniques are integral to clinical practice.

The short-axis out-of-plane ultrasound-guided puncture technique has been extensively adopted for radial artery catheterization due to its benefits, such as the ease of acquiring vascular short-axis images, improved visualization of the anatomical relationship between target vessels and surrounding structures, enhanced positioning accuracy, and simplified operation (8). In this study, two out-of-plane puncture techniques were employed for radial artery catheterization. The results indicated no statistically significant difference in first-attempt puncture success rates between the DNTP and UGDE methods (*P* = 0.98). However, the DNTP group demonstrated a significantly higher success rate for first-attempt cannulation (*P* = 0.049), a longer procedural duration (*P* < 0.001), and a reduced incidence of hematoma and vasospasm (*P* = 0.049) in comparison to the UGDE group. Both DNTP and UGDE techniques employ ultrasound-guided needle insertion, yet they differ in their methodologies: DNTP utilizes dynamic guidance by simultaneously advancing the needle and cannula into the radial artery, while UGDE involves slightly lowering the needle hub after confirming intravascular tip placement, followed by advancing the cannula while gradually withdrawing the needle. This methodological distinction accounts for the comparable first-attempt puncture success rates. DNTP integrates the advantages of short-axis out-of-plane imaging with long-axis in-plane needle tip visualization through the synchronized movement of the probe and cannula, facilitating real-time dynamic monitoring of the needle tip during catheterization and thereby enhancing cannulation success (13–15). The extended procedural time associated with DNTP is attributed to the continuous real-time ultrasound monitoring required until full needle placement, leading to marginally longer operation times compared to UGDE.

Vasospasm and hematoma are prevalent complications associated with radial artery catheterization. Research indicates that repeated punctures at the same site significantly increase the risk of vasospasm (5). Furthermore, factors such as patient anxiety and pain can trigger reflex vasoconstriction upon needle entry into the blood vessel. This reflex not only elevates resistance during needle insertion but also heightens the likelihood of unsuccessful punctures, often necessitating multiple attempts. These repeated attempts can lead to a range of complications, including bleeding and hematoma formation (10, 16–18). The DNTP method offers dynamic guidance for the puncture needle into the radial artery. In the event of arterial spasm during needle insertion, the needle’s position can still be monitored, enabling timely adjustments to maintain the needle tip’s alignment within the artery. This approach prevents the puncture needle from damaging the vessel wall and causing local hematoma, thereby reducing the incidence of complications.

## Conclusion

In summary, the utilization of the DNTP technique under ultrasound guidance for radial artery puncture and catheterization significantly enhances the success rate of first-attempt procedures while concurrently minimizing the incidence of complications associated with repeated punctures. Although these findings support its clinical safety and potential for optimizing subsequent treatments, several study limitations warrant cautious interpretation: First, while the sample size achieved prespecified power for the primary endpoint (puncture time), it was underpowered for secondary outcomes (e.g., complication rates), potentially overlooking clinically significant differences. Second, secondary analyses lacked adjustment for multiple testing, necessitating cautious interpretation of these results. Third, the clinical relevance of reduced puncture time remains incompletely validated due to insufficient correlation with patient-centered outcomes (e.g., shock resolution speed). Finally, the open-label design risked operator-induced performance bias, though independent endpoint adjudication partially mitigated this concern. Future investigations should: (1) expand sample sizes to validate complication rate differences; (2) prespecify shock-related timeliness metrics (e.g., lactate clearance rate dynamics) to establish clinical significance; and (3) implement multicenter analytical platforms to minimize operator-dependent bias and enhance generalizability.

## Data Availability

The original contributions presented in this study are included in this article/Supplementary material, further inquiries can be directed to the corresponding authors.
